# Supplementary material for: Development of a computer-aided design software for the quantitative evaluation of aesthetic damage
Source: PLoS One. 2019 Dec 18;14(12):e0226322. doi: 10.1371/journal.pone.0226322 (PMC6919621; doi:10.1371/journal.pone.0226322)
Supplement: S1 Code site — 1. (ZIP) [file pone.0226322.s001.zip › [EESC_jr][Projeto]Relatorio_07_21/reports/templates_zip/gentelella-master/vendors/select2/docs/index.html]

---
layout: home
title: Select2 - The jQuery replacement for select boxes
slug: home
---

# Select2

The jQuery replacement for select boxes

Download Select2

Currently v4.0.3

{% include notice-previous.html %}

Select2 gives you a customizable select box with support for searching,
tagging, remote data sets, infinite scrolling, and many other highly used
options.

---

#### In your language

Select2 comes with support for
RTL environments,
searching with diacritics and
over 40 languages built-in.

#### Remote data support

Using AJAX you can efficiently
search large lists of items.

#### Fits in with your theme

Fully skinnable, CSS built with Sass and an
optional theme for Bootstrap 3.

#### Fully extensible

The plugin system
allows you to easily customize Select2 to work exactly how you want it
to.

#### Dynamic item creation

Allow users to type in a new option and
add it on the fly.

#### Full browser support

Support for both modern and legacy browsers is built-in, even including Internet Explorer 8.

---

## Getting started with Select2

In order to use Select2, you must include the JavaScript and CSS file on
your website. You can get these files built for you from many different
locations.

### Using Select2 from a CDN

Select2 is hosted on both the
cdnjs and
jsDelivr CDNs, allowing
you to quickly include Select2 on your website.

1. Include the following lines of code in the `<head>`
   section of your HTML.

   {% highlight html %}
   {% endhighlight %}

   Immediately following a new release, it takes some time for CDNs to
   catch up and get the new versions live on the CDN.
2. Initialize Select2 on the `<select>` element that you
   want to make awesome.

   {% highlight html %}
   {% endhighlight %}
3. Check out the examples page to start using
   the additional features of Select2.

### Downloading the code locally

In some situations, you can't use Select2 from a CDN and you must include
the files through your own static file servers.

1. Download the code
   from GitHub and copy the `dist` directory to your project.
2. Include the following lines of code in the `<head>`
   section of your HTML.

   {% highlight html %}
   {% endhighlight %}
3. Check out the examples page to start using
   the additional features of Select2.

## The different Select2 builds

Select2 provides multiple builds that are tailored to different
environments where it is going to be used. If you think you need to use
Select2 in a nonstandard environment, like when you are using AMD, you
should read over the list below.

| Build name | When you should use it |
| --- | --- |
| Standard (`select2.js` / `select2.min.js`) | This is the build that most people should be using for Select2. It includes the most commonly used features. |
| Full (`select2.full.js` / `select2.full.min.js`) | You should only use this build if you need the additional features from Select2, like the compatibility modules or recommended includes like jquery.mousewheel |
